# Supplementary material for: Effects of Dietary Multienzyme Complex Supplementation on Growth Performance, Digestive Capacity, Histomorphology, Blood Metabolites and Hepatic Glycometabolism in Snakehead (Channa argus)
Source: Animals (Basel). 2022 Feb 4;12(3):380. doi: 10.3390/ani12030380 (PMC8833691; doi:10.3390/ani12030380)
Supplement: Supplementary file 1 [file animals-12-00380-s001.zip › animals-1571282-supplementary.pdf]

Supplementary Table S1. Primers used for real -time PCR analysis of snakehead mRNAs

Gene	Forward Primer (5′-3′)	Reverse Primer (3′-5′)	Amplicon Length (bp)
Pepsinogen A	GTCTGGCCTTCCAGAGTATTG	GCTGCTAAGGTAGACAGAGAAC	107
Trypsinogen	GCTCTGTTCGCAGTGGCATTT	GCACTCATAGCCTCCGACAATCT	72
Amylase	CCAGTGTCCCCTATTCCAGTTTG	CTGGCATCACCGTAGTTCTCAAT	85
Hexokinase	ACTCGTCTCCCTCTGGGCTTCACC	CCCTCAACAGTCCCACCACATCTT	135
Fructose1, 6-Bisphosphatase	GGGAAAGCTGCGACTCCTGTATGA	CCGTCTCACTTCTGGGCATGTCTC	203
Glucose-6-phosphatase	CCCTCTTGTGGCTGAGATGTTTGG	GTTCAGCCAGTCTCCAATCACAGC	234
Glycogen synthase	ACCAGCTCACTTCCTTCCTGTTCC	GGCACAGAGGCTGGTCGAGGGTAA	234
18 S	CCCAATATTTCAAACCCAGTCTGT	CCACCGCTAAGAGTTGTCACAGTT	91

Supplementary Table S2. Identification of significantly different metabolites between MEC added and control snakeheads.

Metabolites	RT	Mass	VIP	*P* Value	FC
E1 to Control					
21-hydroxypregnenolone	6.730	91	2.664	0.016	0.403
Norleucine	8.488	86	2.073	0.039	2.829
Dithioerythritol	8.583	73	1.097	<0.001	2.003
uric acid	9.353	171	1.023	0.012	1.883
4-hydroxyphenylacetic acid	9.665	179	1.230	<0.001	0.443
Indolelactate	9.761	130	3.344	0.008	0.101
Proline	10.20	142	1.017	0.011	1.997
Glycine	10.28	174	1.114	<0.001	1.869
N-Methyl-L-glutamic acid	11.72	172	2.241	0.047	2.152
3-hydroxy-L-proline	12.47	68	1.263	<0.001	2.312
aspartic acid	13.03	232	1.062	0.002	1.872
D-alanyl-D-alanine	14.41	188	1.268	0.005	2.336
Taurine	15.43	326	1.147	<0.001	1.881
D-(glycerol-phosphate)	16.67	299	1.171	0.008	2.302
Sorbose	18.52	103	1.078	0.014	0.557
E2 to Control					
5,6-dihydrouracil	6.55	171	3.201	0.019	<0.001
Dithioerythritol	8.583	73	1.024	<0.001	1.996
4-hydroxyphenylacetic acid	9.665	179	1.359	<0.001	0.392
Proline	10.20	142	1.074	0.002	1.853
Glycine	10.28	174	1.014	0.002	1.731
Carbazole	11.36	113	2.636	0.035	2.882
N-Methyl-L-glutamic acid	11.72	172	2.305	0.049	2.073
Aminomalonic acid	12.35	218	1.058	0.034	2.598
Phloroglucinol	14.82	342	3.486	0.001	3.612
Taurine	15.43	326	1.121	<0.001	1.872
Glucoheptonic acid	16.11	73	1.073	<0.001	0.582
Sorbose	18.52	103	1.797	<0.001	0.217
Mannose	19.36	205	1.034	0.042	0.680

uric acid	22.17	441	1.152	0.007	1.786
Pipecolinic acid	23.12	156	2.258	0.030	0.219
beta-Glycerophosphoric acid	23.34	73	1.067	<0.001	1.770
Glutaric Acid	27.05	116	2.332	0.019	0.471
E3 to Control					
Dithioerythritol	8.583	73	1.281	<0.001	2.178
4-hydroxyphenylacetic acid	9.665	179	1.446	<0.001	0.343
Proline	10.20	142	1.065	0.011	1.882
Glycine	10.28	174	1.068	<0.001	1.807
Dihydroxyacetone	12.20	103	4.039	<0.001	<0.001
L-homoserine	14.15	146	2.597	0.039	0.341
Phloroglucinol	14.82	342	3.701	<0.001	5.294
Taurine	15.43	326	1.214	<0.001	1.884
citric acid	17.66	273	1.126	<0.001	0.521
Sorbose	18.52	103	1.932	<0.001	0.148
Uric acid	22.17	441	1.218	0.005	1.864
Pipecolinic acid	23.12	156	3.638	<0.001	0.066
Guanosine	28.71	73	1.254	<0.001	0.421
Gentiobiose	30.12	73	1.210	<0.001	0.466

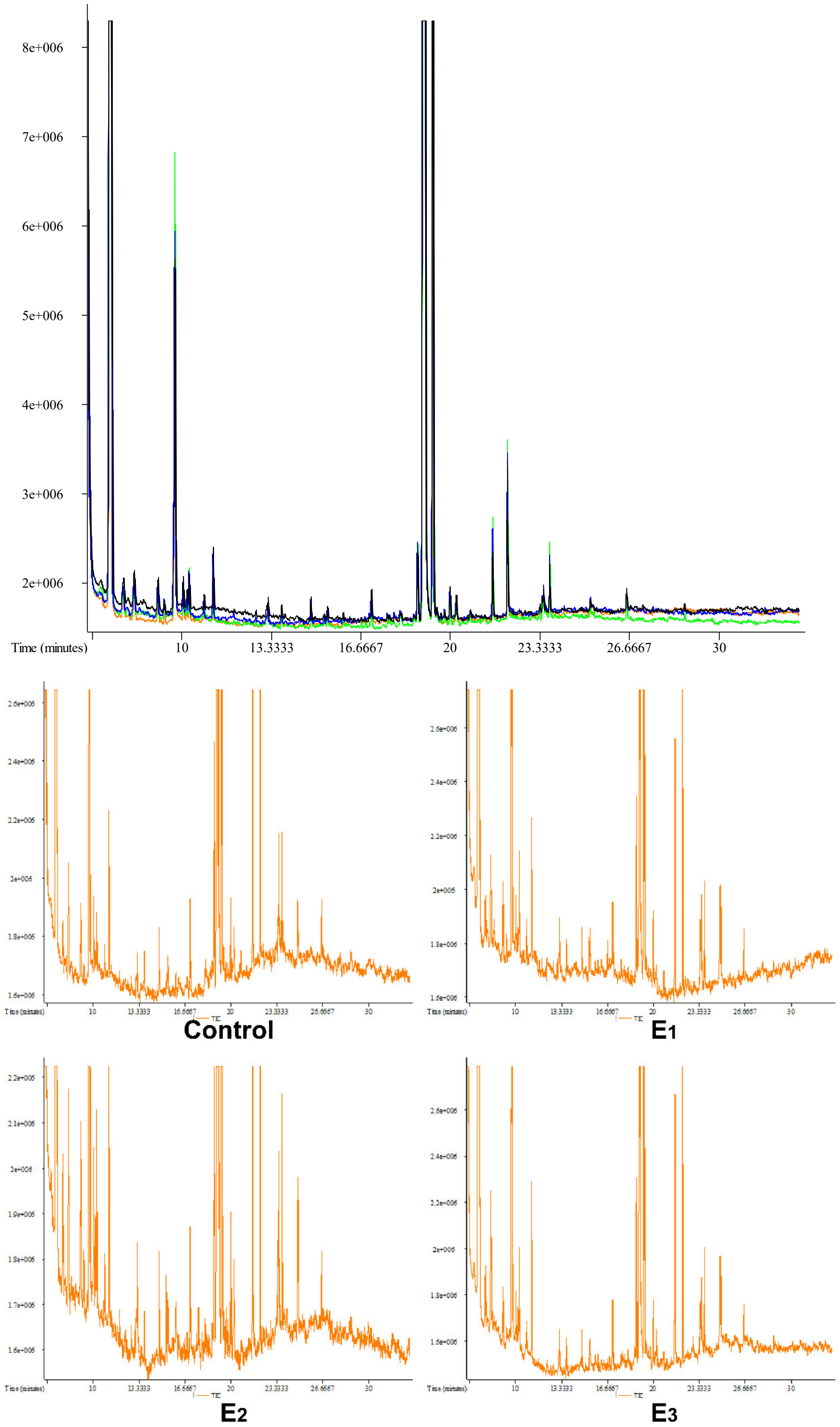

Figure S1. Typical total ion chromatograms (TIC) of the four experimental groups obtained from GC- TOF/MS analysis.
